# Small nucleolar RNA signatures as biomarkers for non-small-cell lung cancer

**DOI:** 10.1186/1476-4598-9-198

**Published:** 2010-07-27

**Authors:** Jipei Liao, Lei Yu, Yuping Mei, Maria Guarnera, Jun Shen, Ruiyun Li, Zhenqiu Liu, Feng Jiang

**Affiliations:** 1 Department of Pathology, University of Maryland School of Medicine, Baltimore, MD. USA; 2 Department of Surgery, University of Maryland School of Medicine, Baltimore, MD. USA; 3 Division of Biostatistics of The University of Maryland Greenebaum Cancer Center, University of Maryland School of Medicine, Baltimore, MD. USA

## Abstract

**Background:**

Non-small-cell lung cancer (NSCLC) is the leading cause of cancer death. Early detection of NSCLC will improve its outcome. The current techniques for NSCLC early detection are either invasive or have low accuracy. Molecular analyses of clinical specimens present promising diagnostic approaches. Non-coding RNAs (ncRNAs) play an important role in tumorigenesis and could be developed as biomarkers for cancer. Here we aimed to develop small nucleolar RNAs (snoRNAs), a common class of ncRNAs, as biomarkers for NSCLC early detection. The study comprised three phases: (1) profiling snoRNA signatures in 22 NSCLC tissues and matched noncancerous lung tissues by GeneChip Array, (2) validating expressions of the signatures by RT-qPCR in the tissues, and (3) evaluating plasma expressions of the snoRNAs in 37 NSCLC patients, 26 patients with chronic obstructive pulmonary disease (COPD), and 22 healthy subjects.

**Results:**

In the surgical tissues, six snoRNAs were identified, which were overexpressed in all tumour tissues compared with their normal counterparts. The overexpressions of the genes in tumors were confirmed by RT-qPCR. The snoRNAs were stably present and reliably detectable in plasma. Of the six genes, three (SNORD33, SNORD66 and SNORD76) displayed higher plasma expressions in NSCLC patients compared with the cancer-free individuals (All < 0.01). The use of the three genes produced 81.1% sensitivity and 95.8% specificity in distinguishing NSCLC patients from both normal and COPD subjects. The plasma snoRNA expressions were not associated with stages and histological types of NSCLC (All > 0.05).

**Conclusions:**

The identified snoRNAs provide potential markers for NSCLC early detection.

## Background

Non-small-cell lung cancer (NSCLC) is the number one cancer killer in the USA and worldwide [1]. The overall 5-year survival rate for stage I NSCLC patients who are typically treated with surgery remain up to 83%. In contrast, only 5-15% and less than 2% of patients with stage III and IV NSCLC are alive after five years [1]. These statistics provide the primary rationale to improve NSCLC early detection. Chest X-ray and plasma cytology have been used for detection of early NSCLC [2,3]. However, the sensitivity was low [2,3]. Although the bronchoscopy excels at detecting centrally occurring lung tumor, it is invasive. CT provides excellent anatomic information and can noninvasively detect lung cancer at small size; however, the improved sensitivity is associated with over-diagnosis [2,3]. Furthermore, it is still unclear whether CT screening can ultimately reduce lung cancer mortality [4]. Therefore, the development of noninvasive approaches that can reliably detect early stage NSCLC is clinically important.

Non-coding RNAs (ncRNAs) are functional transcripts that do not code for proteins, however, play a major role in regulating almost every level of gene expression [5]. In addition to highly abundant and functionally important transfer and ribosomal RNAs, ncRNAs include other RNAs such as small nucleolar RNAs (snoRNAs), microRNAs (miRNAs), short interfering RNAs (siRNAs), piwi-associated RNAs, small Cajal body-specific RNAs (scaRNAs), snRNAs (small nuclear RNAs), and long ncRNAs that are still partially understood [5-8]. Of the small ncRNAs, miRNAs and siRNAs have extensively been studied in carcinogenesis [5-8]. Differential expressions of miRNAs in lung cancer and their potential diagnostic values have been intensively evaluated [9,10]. For instance, abnormal expressions of some miRNAs measured in lung tumor tissues were prognostic factors for overall survival of the patients [11-13]. Furthermore, serum miRNA signatures were identified that can be used to predict outcome of the disease [14]. In addition, we demonstrated that examining altered miRNA expressions in sputum could improve early detection of lung cancer [15,16]. Recently, new and unexpected functions of other types of small ncRNAs have been discovered, revealing that the molecules have highly diverse roles and are actively involved in the processes of carcinogenesis than previously thought [5]. Therefore, investigation of dysregulation of the small ncRNAs in the development and progression of lung tumorigenesis, and their diagnostic values is necessary.

SnoRNAs represent one of the largest groups of functionally diverse trans-acting ncRNAs currently known in mammalian cells [17]. NcRNAs range between 60-150 nucleotides in length [8,17-19]. From a structural basis, snoRNAs fall into two categories termed box C/D snoRNAs (SNORDs) and box H/ACA snoRNAs (SNORAs) [17-19]. SNORDs serve as guides for the 2'-*O*-ribose methylation of rRNAs or snRNAs, whereas SNORAs are guides for the isomerization of uridine residues into pseudouridine [20,21]. Accumulated evidence suggests that snoRNAs can target other RNAs including snRNAs and possibly messenger RNAs [19]. Furthermore, a link between snoRNA and carcinogenesis was first reported by Chang et al, who found that snoRNA h5sn2 was highly expressed in normal brain, but its expression was dramatically reduced in meningioma, suggesting a role for the loss of snoRNA h5sn2 in brain tumorigenesis [21]. Recently, a homozygous 2 bp (TT) deletion in snoRNA U50 was discovered in prostate cancer cell lines and localized prostate tumor tissues, while heterozygous genotype of the deletion occurred more frequently in women with breast cancer [22,23]. Although studies are just emerging, snoRNAs may play malfunction in the development and progression of human malignancy. In this report, we first profiled snoRNA expression signatures of lung cancer tissues and then found that the identified snoRNAs were significantly upregulated in tumor tissues and plasma of NSCLC patients. The snoRNAs might provide potential markers for early detection of NSCLC.

## Results

### Identifying snoRNA signatures whose aberrant expression levels were associated with NSCLC

**Figure 1:**
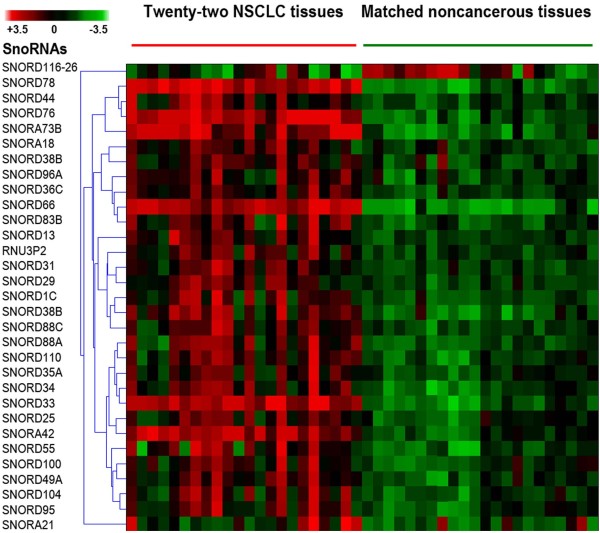
**SnoRNAs differentially expressed in 22 stage I non-small-cell lung cancer (NSCLC) tissues versus normal lung tissues**. Hierarchical clustering of 31 snoRNA genes with a significantly different expression (p < 0.01) in tumour tissues. Rows represent individual genes; columns represent individual tissue samples. The scale stands for the intensity of gene expression (log2 scale ranges between -3.5 and 3.5).

To define and validate snoRNA signatures whose altered expressions were associated with early stage NSCLC, we obtained surgical specimens from 22 stage I NSCLC patients who had either a lobectomy or a pneumonectomy. The cases consisted of 11 patients with squamous cell carcinoma (SCC) and 11 patients with adenocarcinoma (AC) (Table 1). The GeneChipR Arrays comprised probe sets for human snoRNAs, scaRNAs and miRNA coverage of human, mouse, rat, canine, and rhesus macaque was performed on the clinical specimens. We only analyzed and compared expressions of human mature 352 snoRNAs in the tumor and noncancerous tissue specimens. When P value < 0.01 was used as a cutoff, of the snoRNAs analyzed, 30 were overexpressed and one were underexpressed with ≥ 1.0 fold-change in lung NSCLC tissues compared with the corresponding noncancerous lung tissues (P < 0. 01) (Fig. 1 and Additional Table 1). Using a predefined criterion of a change ≥1.5-fold, we identified six snoRNAs that were statistically differently expressed between the paired tumor and noncancerous samples (all P < 0.01). These included SNORD33, SNORD66, SNORD73B, SNORD76, SNORD78, and SNORA42 (Additional Table 1). Furthermore, there was no statistical difference of expressions of the six genes between AC and SCC of the lungs, suggesting that the snoRNAs were shared in the two major histological types of NSCLC. More importantly, the six snoRNAs were overexpressed in all 22 NSCLC tissues compared with the paired noncancerous specimens, thus were proceed to the next phase of the study.

**Table 1:** Characteristics of 22 NSCLC patients

	11 SCC (%)	11 AC (%)
Age	68 (SD 13.6)	67 (SD 12.8)
Sex		
Female	3 (27.3)	3 (27.3)
Male	8 (72.7)	8 (72.7)
Race		
White	6 (54.6)	6 (54.6)
African American	5 (45.4)	5 (45.4)
Smoking status		
Pack-years	33.2 (SD 20.9)	27.2 (SD 18.3)
Stage		
All are stage I	11	11

Abbreviations: NSCLC, non-small-cell lung cancer; SCC, squamous cell carcinoma; AC, adenocarcinoma; SD, standard deviation.

### Validating expressions of the six snoRNAs on surgical tissues by RT-qPCR

To determine whether the data derived from the microarray platform could be confirmed by different technique, the expressions of the six snoRNAs were assessed by using RT-qPCR assay in the same 22 NSCLC tissues and paired noncancerous lung specimens. More than 1.5-fold overexpression of the six snoRNAs was found in all NSCLC cases, respectively, as compared with that in the corresponding noncancerous tissues (All P < 0.0001). Pearson test showed that each gene's the relative expression level by RT-qPCR and log 2-transformed signal intensity value by microarray significantly correlated (Additional Table 2). The observations suggested that the identified snoRNAs could be confirmed by a different technique, and thus the differential expressions of the identified genes in the surgical specimens were based on accurate quantification. Furthermore, no significant expression difference of the six snoRNAs by RT-qPCR (P > 0.05) was observed for the stage I tumor samples with different histological types. The observation provides further evidence that the elevated snoRNA expressions in cancer tissues are not histologically specific changes.

### Quantifying snoRNA expression in plasma by RT-qPCR assay

Although previous reports have documented presence of detectable quantifies of miRNA in plasma, whether snoRNAs stably exist in the cell free body fluid remain unknown. To determine if the snoRNAs were present in plasma, we first prepared two RNA pools containing equal amounts of RNA from plasma of five cancer patients and five healthy individuals, respectively. Expression of each snoRNA was then measured by RT-qPCR in the pooled RNA samples. All tested snoRNAs had ≤ 32 Ct values in both pools, indicating that the snoRNAs existed in plasma. To determine if the snoRNAs were stably and reliably detected in plasma, we first assessed the stability of endogenous ncRNAs in plasma, because it contains high levels of RNase activity. Plasma obtained from three healthy subjects was split into two parts, respectively. One part of each sample was treated with Ribonuclease A at final concentration of 10 μg/mL, whereas the second part was not added with Ribonuclease A. Expressions of the six snoRNAs and a miRNA, miR-21, were measured by using RT-qPCR in parallel. The abundance of all snoRNAs and the miR-21 in the plasma samples with the different treatments was fairly equable (P > 0.05) (Additional Fig. 1), demonstrating that the plasma snoRNAs were resistant to RNase digestion. To further verify the stability of the plasma snoRNAs, plasma was collected from another three healthy individuals. Each sample was divided into 4 parts. The first aliquot from each specimen was processed immediately for isolating RNA, while others were stored in -80°C and processed for RNA isolation on day 1, 7 and 30. Expressions of the snoRNAs were measured at the same time in these specimens that were processed from the different time points. Expression level of miR-21 was also simultaneously assayed on the specimens. Each of the snoRNAs and miR-21 displayed equal expression levels between the samples, respectively (Additional Fig. 2). Therefore, like miRNA, snoRNAs are present in a stable form and consistently measurable in archived plasma samples.

To determine specificity of snoRNA quantification by RT-qPCR assay in plasma, SNORD76 and SNORD78 that are located in the same chromosomal region (1q25.1), were synthetically generated (Integrated DNA Technologies, Inc, Coralville, IA). Each one subjected to two independent RT-qPCR reactions, where in each reaction there were present PCR primers specific to only one of the two genes. Amplification only of the appropriate gene matching the specific primer was observed, indicating that RT-qPCR assay could detect snoRNA with high specificity. Furthermore, given that the average size of snoRNAs (70 to 90 nt) was longer than that of miRNAs (22 nt), we evaluated whether the RT-qPCR assay would only detect snoRNA but not DNA sequences. RNA preparation extracted from two plasma samples was divided into two identical portions, which were then treated with or without DNase I Reaction Buffer, respectively. The expression level of the snoRNAs in each of the two groups was quantified by RT-qPCR. There was no difference in the gene expression levels between RNA extracted from the samples treated with DNase I and RNA from the samples without treatment (P < 0.05). The result suggests that the snoRNAs could be specifically detected without contaminating genomic DNA in RNA preparations.

To determine the sensitivity of detecting snoRNA by RT-qPCR assay in plasma, the total RNA was isolated from plasma of three healthy subjects and then diluted in diethyldicarbonate water by ten orders of magnitude, respectively. The serially diluted RNAs served as experimental samples for measuring expression of the snoRNAs. The results showed excellent linearity between the RNA input and the Ct values for RT-qPCR. Furthermore, the assay had a dynamic range of at least six orders of magnitude (R2 = 0.997), and was capable of detecting al least 100 copies of the target snoRNA genes. Altogether, the snoRNAs could be accurately and reliably measured in blood plasma by the RT-qPCR platform.

### Evaluating plasma expressions of the six snoRNAs in NSCLC patients, COPD patients, and healthy individuals

**Figure 2:**
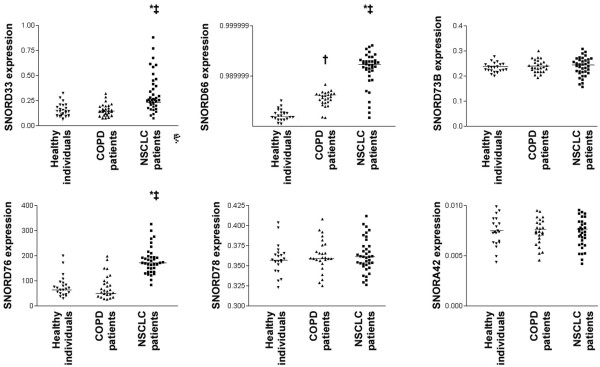
**Plasma expression levels of the six snoRNAs in 22 healthy controls, 26 patients with chronic obstructive pulmonary disease (COPD), and 37 patients with non-small-cell lung cancer (NSCLC)**. Horizontal lines denote mean values. *, statistical significance (< 0.01) of expression levels of snoRNA between NSCLC patients and healthy controls. ^†^, statistical significance (< 0.01) of expression levels of snoRNA between COPD patients and healthy controls. ^‡^, statistical significance (< 0.01) of expression levels of snoRNA between NSCLC patients and COPD patients.

RT-qPCR assay was successfully performed in all plasma samples of 37 NSCLC patients and cancer-free individuals including 26 patients with chronic obstructive pulmonary disease (COPD) and 22 healthy subjects (Table 2, Fig. 2). Of the six genes, SNORA42, SNORD73B, and SNORD78 showed measurable plasma expressions, which, however, did not significantly differ between NSCLC patients, COPD patients, and healthy individuals (Table 3, Fig. 2). In contrast, SNORD33, SNORD66, and SNORD76 exhibited significantly higher expressions in NSCLC patients as compared with healthy controls, suggesting that the elevated expressions of the three genes in plasma might be cancer-associated changes (All P < 0.01). Among the three cancer-associated genes, SNORD66 was also considerably overexpressed in plasma of COPD patients as compared with healthy controls (P < 0.01), implying that the cancer-associated gene whose changes were also related to COPD. However, when plasma expression of SNORD66 was compared between NSCLC and COPD groups, it was significantly higher in the NSCLC patients as compared with the COPD patients (P < 0.01) (Table 3, Fig. 2). Therefore, although displaying higher plasma level in COPD patients as compared with healthy subjects, SNORD66 had lower plasma expression level in COPD patients as compared with NSCLC patients. On the contrary, SNORD33 and SNORD76 showed significantly higher expressions in plasma of the NSCLC group as compared with COPD group (All P < 0.01), whereas there was no statistical difference regarding expressions of the two genes between COPD and healthy groups (All P > 0.05; Table 3, Fig. 2). The finding that the increased plasma expressions of SNORD33 and SNORD76 solely occurred in NSCLC patients suggest that these two genes whose upregulations could be cancer-specific changes. Taken together, the three snoRNAs might provide potential biomarkers in distinguishing NSCLC patients from both healthy subjects and COPD patients. Therefore, the three genes, SNORD33, SNORD66, and SNORD76, proceed to the next step of the study.

**Table 2:** Characteristics of a cohort of NSCLC patients, healthy individuals, and COPD patients

	37 NSCLC (%)	22 healthy individuals (%)	26 COPD patients (%)
Age*	68 (53-75)	64 (48-69)	67 (56-72)
Sex			
Female	11 (29.73)	7 (31.82)	8 (30.77)
Male	26 (70.27)	15 (68.18)	18 (29.23)
Race			
White	22 (59.46)	13 (59.09)	16 (61.54)
African American	15 (40.54)	9 (40.91)	10 (38.55)
Smoking status	38 ± 27 (pack-years)	33 ± 29 (pack-years)	37 ± 25 (pack-years)
Histological types			
SCC	16 (43.24)		
AC	21 (56.76)		
Stage			
I	10 (27.03)		
II	12 (32.43)		
III-IV	15 (40.54)		

Abbreviations: NSCLC, non-small-cell lung cancer; COPD, chronic obstructive pulmonary disease; SCC, squamous cell carcinoma, AC, adenocarcinoma.

*Data are presented as median (range).

**Table 3:** Plasma expression levels of snoRNAs

SnoRNAs	Twenty-two healthy individuals	Twenty-six COPD patients	Thirty-seven NSCLC patients
	**Mean ± SD**	**Mean ± SD**	**Mean ± SD**

SNORD33	0.1275 ± 0.0631	0.1368 ± 0.0577	0.2466 ± 0.1907 *^‡^
SNORD66	0.9821 ± 0.0011	0.9857 ± 0.0015^†^	0.9923 ± 0.0035 *^‡^
SNORD73B	0.2325 ± 0.0179	0.2297 ± 0.0248	0.2405 ± 0.0363
SNORD76	70.7031 ± 42.2108	64.2773 ± 47.7285	173.1275 ± 52.7852 *^‡^
SNORD78	0.3571 ± 0.0186	0.3598 ± 0.0198	0.3613 ± 0.0208
SNORA42	0.0074 ± 0.0016	0.0075 ± 0.0016	0.0076 ± 0.0017

Abbreviations: COPD, chronic obstructive pulmonary disease; NSCLC, non-small-cell lung cancer; SD, standard deviation.

* Statistical significance of expression levels of the snoRNAs between NSCLC patients and healthy controls. P < 0.01.

^† ^Statistical significance of expression levels of the snoRNA between COPD patients and healthy controls. P < 0.01.

^‡ ^Statistical significance of expression levels of the snoRNAs between NSCLC patients and COPD patients. P < 0.01.

### Evaluating diagnostic efficiency of the snoRNAs in plasma for NSCLC

Receiver-operator characteristic (ROC) analyses were applied to evaluate the capability of using the three snoRNAs to discriminate NSCLC patients from healthy individuals and COPD patients. As shown in Table 4, the individual snoRNAs exhibited area under the receiver-operator characteristic (AUC) values of 0.8064-0.8233 in distinguishing NSCLC patients from healthy subjects. When optimum cutoffs were selected at 0.2262, 0.9893, and 133.6453, the snoRNAs yielded 72.65-75.36% sensitivity and 85.02-87.66% specificity. Furthermore, the individual snoRNAs exhibited 0.7903-0.8186 AUCs in distinguishing NSCLC patients from COPD patients (Table 4). When optimal cutoffs were chosen at 0.2278, 0.9902, and 136.1379, the three genes produced 71.46.3-73.98% sensitivity and 84.91-87.58% specificity in indentifying NSCLC patients from COPD patients (Table 4).

**Table 4:** Capability of the three snoRNA genes to discriminate NSCLC patients from healthy controls and COPD patients*

	Distinguishing NSCLC from healthy subjects				Distinguishing NSCLC from COPD			
	
SnoRNAs	AUC (SE)	Thresholds	Sensitivity	Specificity	AUC (SE)	Thresholds	Sensitivity	Specificity
			%	%			%	%
SNORD33	0.8233 (0.06)	0.23	72.97	86.36	0.82 (0.05)	0.23	72.97	84.62
SNORD66	0.8139 (0.046)	0.99	75.68	77.27	0.7903 (0.04)	1.00	72.97	80.77
SNORD76	0.8064 (0.03)	133.65	70.27	90.91	0.8149 (0.06)	136.14	70.27	88.46
The three genes used in combination	0.89 (0.06)		83.78	95.45	0.88 (0.06)		81.08%	96.15%

* Receiver-operator characteristic (ROC) curve and the area under ROC curve (AUC) analyses were applied to determine optimal thresholds that define expression levels of the tested genes, yielding corresponding maximum sensitivity and specificity of each gene in diagnosis of NSCLC from either healthy or COPD individuals.

Abbreviations: NSCLC, non-small-cell lung cancer; COPD, chronic obstructive pulmonary disease; SD, standard deviation; SE, standard error.

We then evaluated the combination of the three snoRNAs for identification of NSCLC. The three snoRNAs in combination produced 0.8935 AUC, being considerably higher than those of each individual gene (All P < 0.05) in identification of NSCLC group from healthy group (Table 4). Accordingly, given a specificity of 95.5%, the composite panel of the snoRNAs revealed a sensitivity of 83.8% in detection of NSCLC. The parameters were significantly higher compared with those of an individual snoRNAs (all p < 0.05) in distinguishing cancer patients from normal controls. Furthermore, the three genes used in combination created 0.8827 AUC in distinguishing cancer patients from COPD individuals, yielding 81.1% sensitivity and 96.2% specificity in detection of NSCLC patients. In addition, when the optimal cutoff for each gene that was selected to discriminate NSCLC group from COPD group was used to identify cancer patients from healthy controls, ROC analyses showed that the three genes in combination revealed 83.8% sensitivity and 96.2% specificity. The results, therefore, documented that the combined analysis of the three genes in a panel had a reasonable power to differentiate NSCLC patients from both COPD patients and healthy controls.

Spearman rank correlation analysis indicated that the estimated correlations among expression levels of the three snoRNAs in plasma were low (all R^2 ^< 0.50, P > 0.05). The data suggested that expressions of the snoRNAs were complementary to each other, and further supported that the combined analyses of the genes outperformed a single one used alone. Therefore, the panel of the snoRNAs provides a reasonable power for the early detection of NSCLC in plasma.

Moreover, the composite use of the three snoRNAs had no statistical differences of sensitivity and specificity between different stages of NSCLC (P > 0.05) (Additional Tables 3). The finding that altered expressions of the snoRNAs are found not only in advanced stage, but also in early stage NSCLC might be an important characteristic if they are to be employed for early detection. Additionally, there were no significant differences of sensitivity and specificity for the panel of snoRNAs in discriminating lung SCC and AC patients from healthy controls (P > 0.05) (Additional Tables 3). Therefore, the combined analysis of the three genes in plasma had equal diagnostic efficiency for the two major histological types of NSCLC. There was no association of the changes of the three genes with the age, gender, ethnic, and smoking packer-year of the participants (All p > 0.05) (Additional Tables 4).

## Discussion

In the present study, we profiled snoRNA expression signatures of early stage NSCLC by performing microarray analysis on surgical tissues. Aberrant expressions of the identified signatures were well confirmed by RT-qPCR assay. To the best of our knowledge, this is the first study to globally analyze snoRNA expression patterns in human tumor tissues. Furthermore, we demonstrated that like miRNAs, snoRNAs remained intact and were readily detectable in plasma by using RT-qPCR. More importantly, based on the discoveries, we developed a panel of plasma-based snoRNAs as potential biomarkers for early stage NSCLC. Our data might provide compelling evidence that dysregulations of the snoRNAs could play an important role in lung tumorigenesis, and measuring plasma snoRNAs might serves as a potential noninvasive approach to improve diagnosis of NSCLC.

Although rarely being reported, malfunction of some snoRNAs have recently been considered to contribute to carcinogenesis. For instance, adeno-associated viruses integrated their genome into mouse genome, causing liver cancer [24]. Interestingly, the integration sites identified in the tumors were all located within a DNA interval encoding snoRNAs [24]. Furthermore, the accumulation of gas5-generated snoRNAs was associated with an arrest of cell growth [25], and dysregulations of the snoRNAs were related to growth arrest of breast cancer cells [26,27]. In addition, although snoRNAs and miRNAs are generated by different cellular pathways and function in different cellular compartments, some members of these two types of ncRNAs display numerous genomic similarities [28-30]. Indeed, a number of human snoRNAs with miRNA-like processing signatures were recently identified [31]. The findings were consistent with those in another report [32], in which, a set of miRNAs display functional snoRNA characteristics, and the miRNAs might evolve from snoRNAs [32]. Therefore, some small ncRNAs in human cells that originate from snoRNAs were proposed to function like miRNAs [32]. Moreover, snoRNAs could play a role in posttranscriptional gene silencing. For example, HBII-52, a human SNORD gene, can regulate splicing of serotonin receptor 2C messenger RNA [33]. Like miRNAs, some snoRNAs are located at a chromosomal breakpoint involved in human carcinogenesis. For example, U50 snoRNA was originally discovered from the breakpoint of chromosomal translocation t (3,6) (q27;q15), which was involved in human B-cell lymphoma [34]. It have been suggested that the genes that are frequently located at chromosomal genomic amplification regions might have oncogenic function involved in the promotion of cancer [35-37]. Notably, all the snoRNAs identified in the present study displayed up-regulation in lung tumor specimens. Interestingly, the snoRNAs are located in commonly frequent genomic amplified regions in lung cancer [38,39]. SNORD33 is located in chromosome 19q13.3 that contain potential oncogenes in lung cancer [36,37], while SNORD66 and SNORD76 are situated in chromosomal regions 3q27.1 and 1q25.1, respectively. 3q27.1 and 1q25.1 are two of the most frequently amplified chromosomal segments in solid tumors, particularly NSCLC [35-39]. Therefore, upregulation of the snoRNAs in lung cancer might have oncogenic functions in the cacinogensis. Our primary goal of the current study is marker development. The biological relevance of the snoRNAs in tumorigenesis is currently being investigated at our laboratory.

Most of the previously identified lung cancer associated molecular genetic changes were related to the smoking status. Furthermore, some of the changes were associated with lung inflammatory diseases, especially COPD [40]. The use of such molecular alterations as biomarkers will produce false positive diagnostic rate, thus impeding their future application in clinical settings for diagnosis of lung cancer. The snoRNAs identified from the present research is fairly encouraging as biomarkers, because they highly express in plasma independently of participants' age, gender, ethnic subgroup, and smoking packer-year. In particular, high expressions of SNORD33 and SNORD76 were only observed in plasma from cancer patients. Furthermore, although SNORD66 displayed increased expression in plasma of COPD patients as compared with that in plasma of the healthy controls, it had considerably higher plasma expression level in NSCLC patients compared with COPD individuals. The observation suggests that the snoRNA panel could serves as useful biomarkers in differentiating NSCLC patients from not only healthy individuals, but also COPD subjects. Nonetheless, futures studies to comprehensively investigate biological relevance of the dysregulated SNORD66 in COPD are needed. Moreover, no significant difference regarding plasma expression of the genes was observed at different stages of NSCLC, implying that the potential markers were not stage-specific. In addition, the elevated plasma expression levels of the snoRNAs had equal frequency between AC and SCC of the lungs, suggesting that the genetic changes might be useful biomarkers for the two major histological types of lung cancer.

Although the results look promising, the sensitivity (81.1%) and specificity (95.5%) of the snoRNAs are still not yet efficient for routine clinical application. To surmount the problem, we need to identify additional cancer-associated ncRNAs that can be added to the current ones so that the diagnostic efficacy of the plasma-based approach could be improved. The fundamental mechanism supports this premise is that although only about 352 snoRNAs were analyzed, more than 500 snoRNAs might exist in the human genomic sequences [41]. Furthermore, we expect to improve such result through combing lung cancer-associated miRNAs with the identified snoRNAs [11,13,14]. In addition, other types of ncRNAs, such as piwi-associated RNAs may also play important role in carcinogenesis [5,42]. Including other classes of small ncRNAs with high associated with NSCLC would also improve diagnostic accuracy of the noninvasive approach. To that end, we are analyzing tumor specimens by applying microarray platform to target various types of ncRNAs to develop additional markers for NSCLC.

## Conclusions

We have defined and developed a panel of snoRNAs, whose altered expressions were associated with early stage NSCLC. We demonstrated that the snoRNAs existed in a stable form and were reliably measurable in plasma. Detection of the class of ncRNAs in plasma could potentially be used as a noninvasive diagnostic tool for early NSCLC. Nonetheless, a large multi-center clinical project to further validate the full utility is required before it could be adopted in routine clinical setting.

## Methods

### Patients and clinical specimens

To define and validate snoRNA signatures whose altered expressions were associated with early stage NSCLC, surgical specimens were obtained from 22 lung cancer patients who had either a lobectomy or a pneumonectomy between March 6, 2000 and June 23, 2003 at the University of Maryland Medical Center. All cases were diagnosed with histologically confirmed stage I NSCLC, comprising 11 patients with SCC and 11 patients with AC (Table 1). Tumor tissues were intraoperatively dissected from the surrounding lung parenchyma; paired noncancerous lung tissues were obtained from the same patients at an area distant from their tumors. Serial cryostat sections from the specimens were stained with hematoxylin and eosin to confirm the diagnosis based on the most recent WHO classification of tumors of the lungs [43,44]. None of the patients had received preoperative adjuvant chemotherapy or radiotherapy. To evaluate expressions of the snoRNAs in plasma, 37 NSCLC patients, 22 healthy subjects, and 26 patients with COPD were recruited (Table 2). Peripheral blood was drawn in EDTA tubes (Greiner Bio-One GmbH, Monroe, NC) from the participants and processed within 1 hour of collection by centrifugation at 1,500 × g for 15 min at 4°C. Blood plasma was then collected as previously described [14], and immediately aliquoted and stored in a dedicated -80°C freezer. The study was approved by Institutional Review Board.

#### RNA isolation

Total RNA containing small RNA was extracted from the tissue and plasma specimens by using a mirVana ncRNA Isolation Kit (Ambion, Austin, TX) as previously described [14,16]. The purity and concentration of RNA were determined from OD260/280 readings using a dual beam UV spectrophotometer (Eppendorf AG, Hamburg, Germany). RNA integrity was determined by capillary electrophoresis using the RNA 6000 Nano Lab-on-a-Chip kit and the Bioanalyzer 2100 (Agilent Technologies, Santa Clara, CA). Only RNA extracts with integrity number values > 6 underwent in further analysis.

#### SnoRNA profiling of surgical resected lung tissues

SnoRNA profiling was performed by using GeneChipR Array (Affymetrix, Inc, Santa Clara, CA). The array comprised 7,815 probe sets that were designed to analyze small non-coding RNAs. Microarray experiments were done with all 22 matched malignant and noncancerous sample pairs according to the manufacturer's instructions as described in our previous report [45]. Briefly, 3 μg total RNA was labeled with Biotin FlashTag Biotin Labeling Kit (Affymetrix, Inc). The labeling reaction was hybridized on the arrays in Affymetrix Hybridization Oven 640 (Affymetrix, Inc) at 48°C for 16 hours. The arrays were stained with Fluidics Station 450 using fluidics script FS450_0003 (Affymetrix, Inc), and then scanned on a microarray scanner (Axon Instruments Inc, Foster City, CA). SnoRNA probe outliers were defined as per the manufacturer's instructions (Affymetrix, Inc) and further analyzed for data summarization, normalization, and quality control by using the web-based QC Tool software (http://www.affymetrix.com). The normalized microarray data underwent further analysis as described in statistical section.

#### Quantification of snoRNA expression by real-time RT-qPCR

Expressions of the identified snoRNAs were first validated in the surgically resected tissues and then tested in plasma by using real-time SYBR green RT-qPCR assay. Briefly, 10 ng of plasma RNA was polyadenylated by poly(A) polymerase and reverse transcribed to cDNA using miScript Reverse Transcription kit (Qiagen, Valencia, CA) according to the manufacturer's instructions. RT-qPCR was performed using miScript SYBR Green PCR kit (Qiagen) with the manufacturer provided miScript Universal primer and the snoRNA-specific forward primers in ABI PRISM 7900 Real-time PCR system (Applied Biosystems, Foster City, CA). The primers were designed based on the snoRNA sequences obtained from the gene database of The National Center for Biotechnology Information. The primer sequences for the snoRNAs and their amplification profile are available upon request. Each PCR reaction was carried out in a volume of 25 μl containing 2 μl of the cDNA, 0.1 *μ*mol/l of each primer and 2× SYBR Green PCR Master mix (Qiagen). At the end of the PCR cycles, melting curve analyses were performed. All assays were performed in triplicates, and one no-template control and two interplate controls were carried along in each experiment. Expression levels of the snoRNAs were calculated using comparative cycle threshold (Ct) method as previously described [15,16]. Ct values of the target snoRNAs were normalized in relation to that of small nuclear U6 RNA. U6 RNA was proven as an internal control for ncRNA quantification [15,16,45,46]. ΔCt was calculated by subtracting the Ct values of U6 from those of the snoRNA tested, and fold-change of each snoRNA was determined by the equation 2-ΔΔCt.

#### Statistical analysis

To find snoRNAs that were differentially expressed between paired NSCLC specimens and corresponding noncancerous tissues, we first analyzed the normalized microarray data by using GenePattern (http://www.broad.mit.edu), BRB-ArrayTools version 3.6 (http://linus.nci.nih.gov/BRB-ArrayTools.html), and microarray software suite 4 (TM4) (http://www.tm4.org). We then performed tree visualization by using Java Treeview 1.0 (Stanford University School of Medicine, Stanford, CA). Pearson's correlations were used for the comparison of RT-qPCR and microarray data. ROC curve analysis was done using plasma expression level of each gene for the NSCLC patients, COPD patients, and normal controls by Analyse-it software (Analyse-it Software Ltd, Leeds, UK). Using this approach, AUC identified optimal sensitivity and specificity levels at which to distinguish NSCLC patients from healthy individuals or COPD patients, and corresponding thresholds were calculated for each snoRNA. In addition, Spearman rank correlation was carried out to analyze the correlation between the expressions of the identified snoRNAs. Moreover, the associations between the expression levels of the snoRNAs and both clinicopathologic and demographic characteristics of the cases and controls were evaluated by using univariate and multivariate logistic regression models. All P values shown were two sided, and a P value of < 0.05 was considered statistically significant.

## Competing interests

The authors declare that they have no competing interests.

## Authors' contributions

FJ designed the study, performed research, analyzed data and wrote the paper. JL, YM, JS, and LY performed microarray and RT-qPCR analyses. RL performed analyzed data and wrote the paper. ZL performed data mining analyses. MG recruited patients, obtained written consents, and collected clinical data. All authors have read and approved the final manuscript.

## Supplementary Material

Additional file 1 **SnoRNAs that show changes in clinical specimens of lung cancer patients**. SnoRNAs differentially expressed in non-small cell lung cancer tissues versus normal lung tissues and plasma of cancer patients and control subjects. Click here for file
