# Supplementary material for: Small nucleolar RNA signatures as biomarkers for non-small-cell lung cancer
Source: Mol Cancer. 2010 Jul 27;9:198. doi: 10.1186/1476-4598-9-198 (PMC2919450; doi:10.1186/1476-4598-9-198)
Supplement: Additional file 1 — SnoRNAs that show changes in clinical specimens of lung cancer patients. SnoRNAs differentially expressed in non-small cell lung cancer tissues versus normal lung tissues and plasma of cancer patients and control subjects. [file 1476-4598-9-198-S1.DOC]

**Additional file**

| **Additional Table 1.** Annotated snoRNAs differentially expressed in non-small cell lung cancer tissues versus normal lung tissues | | |
| --- | --- | --- |
| Official gene symbol | Log2 (fold) | P-value |
| SNORD116-26 | -0.717639864 | 0.00135926 |
| SNORA18 | 0.591023455 | 7.2502E-06 |
| SNORD35A | 0.596090591 | 0.00017517 |
| SNORD25 | 0.600807636 | 5.5462E-05 |
| SNORD38B | 0.611615364 | 1.5545E-05 |
| SNORD29 | 0.612612227 | 5.766E-06 |
| RNU3P2 | 0.636824091 | 5.9288E-06 |
| SNORD100 | 0.637317636 | 0.00023909 |
| SNORD49A | 0.642720364 | 8.5187E-06 |
| SNORD96A | 0.648474909 | 3.3282E-05 |
| SNORD95 | 0.6599225 | 0.00026271 |
| SNORD31 | 0.660555409 | 3.1588E-06 |
| SNORD36C | 0.674893409 | 1.1604E-07 |
| SNORD88C | 0.676960955 | 3.4137E-06 |
| SNORD1C | 0.702872545 | 0.00013523 |
| SNORD44 | 0.7168185 | 3.1029E-05 |
| SNORD13 | 0.724133909 | 2.8796E-06 |
| SNORA21 | 0.728817136 | 0.00234082 |
| SNORD104 | 0.731907591 | 9.5676E-05 |
| SNORD110 | 0.799712136 | 2.63E-06 |
| SNORD88A | 0.840233455 | 4.6717E-06 |
| SNORD34 | 0.843215682 | 2.9684E-06 |
| SNORD83B | 1.1505305 | 1.2246E-05 |
| SNORD38B | 1.262247864 | 2.3759E-07 |
| SNORD55 | 1.354700227 | 1.7989E-05 |
| **SNORA42** | **1.569662273** | **4.50E-06** |
| **SNORD76** | **1.886082318** | **6.0336E-07** |
| **SNORD33** | **2.226385409** | **1.1255E-07** |
| **SNORA73B** | **2.282599455** | **4.8661E-07** |
| **SNORD78** | **2.689525273** | **7.5221E-08** |
| **SNORD66** | **3.456219136** | **1.62E-07** |
| The six snoRNAs with average fold change ≥1.5 are given in bold type. | | |

| **Additional Table 2.** Correlation between determinations of snoRNA expressions by microarray and RT-qPCR * | | |
| --- | --- | --- |
| SnoRNAs | Coefficient | P |
| SNORD33 | -0.8938 | 0.0076 |
| SNORD66 | -0.8594 | 0.0053 |
| SNORD73B | -0.8648 | 0.0068 |
| SNORD76 | -0.9753 | 0.0032 |
| SNORD78 | -0.8523 | 0.0095 |
| SNORA42 | -0.9654 | 0.0029 |
| * The Correlation was analyzed by using Pearson's correlations and p-values were obtained using Fisher's z-transformation. | | |

**Additional Fig. 1**


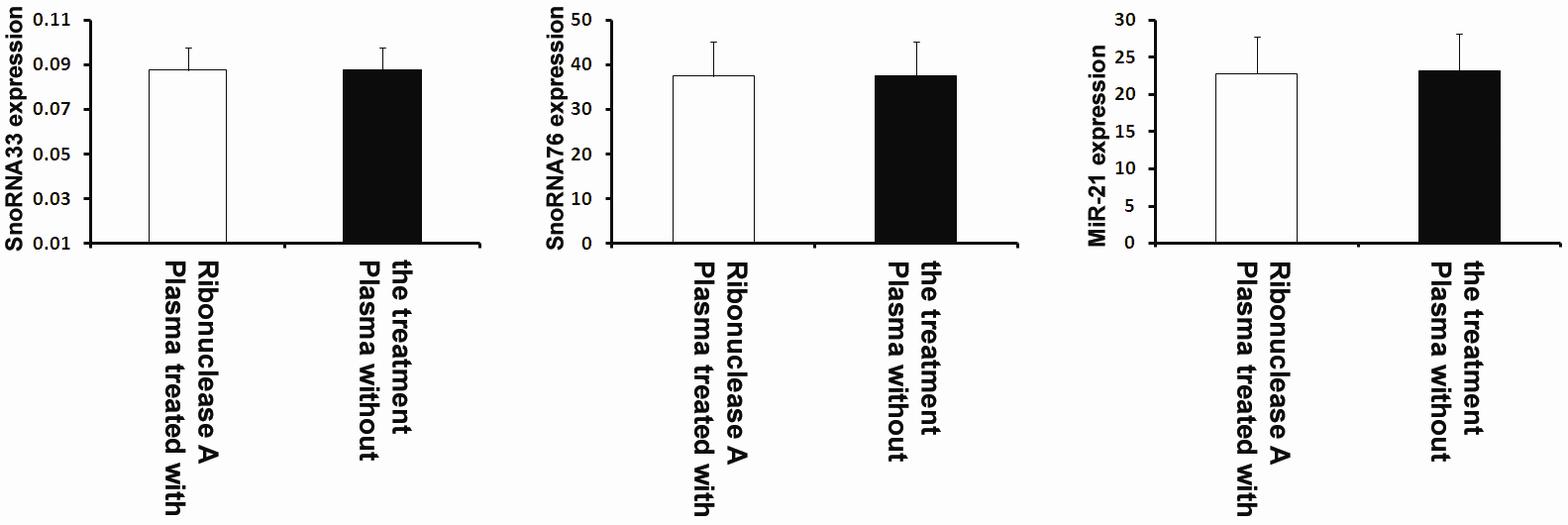


**The stability of endogenous ncRNAs in plasma.**

Plasma obtained from three healthy subjects was split into two parts, respectively. One part of each sample was treated with Ribonuclease A, whereas the second part was not added with Ribonuclease A. Expressions of the snoRNAs and a miRNA, miR-21, were measured by using RT-qPCR in parallel. The abundance of all snoRNAs and the miR-21 in the plasma samples with the different treatments was equable (P>0.05). Therefore, like miRNA, the plasma snoRNAs are resistant to RNase digestion. All six snoRNAs were tested in all the samples. All assays were performed at least three times. The supplementary Fig. 1 only shows the results of SNORD33, SNORD76.

**Additional Fig. 2**


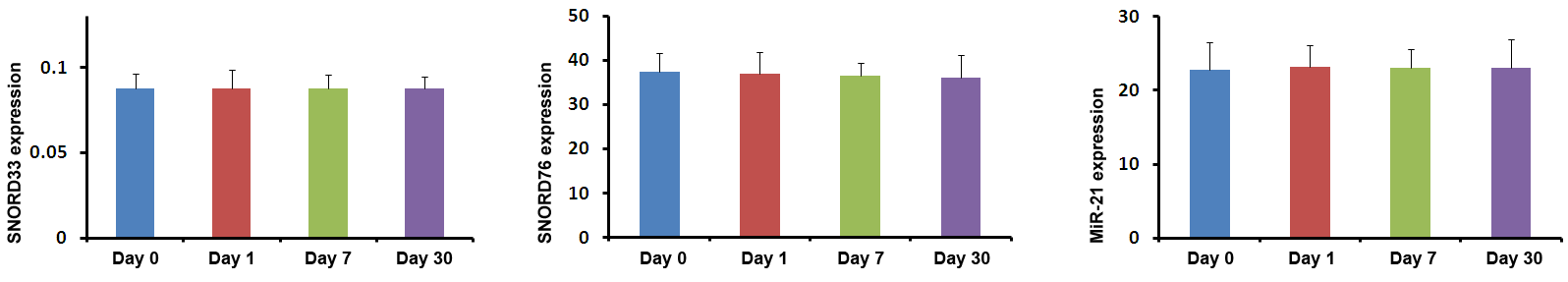


**The expression levels of each snoRNA measured by real-time RT-PCR in the specimens do not change after 30-day storage.**

Plasma obtained from three healthy subjects, and each sample was divided into 4 parts. The first aliquot from each specimen was processed immediately for isolating RNA, while others were stored in -80°C and processed for RNA isolation on day 1, 7 and 30. Expressions of the snoRNAs were measured the same time in these specimens that were processed from the different time points. Expression level of miR-21 was also simultaneously assayed on the specimens. Each of the snoRNAs and miR-21 displayed equal expression levels between the samples. Therefore, like miRNA, snoRNAs are present in a stable form and consistently measurable in archived plasma samples. All six snoRNAs were tested in all the samples. All assays were performed at least three times. The supplementary Fig. 2 only shows the results of SNORD33, SNORD76.

| **Additional Table 3.** Diagnosis of NSCLC patients from cancer-free subjects by the three snoRNAs ^*^ | | | |
| --- | --- | --- | --- |
|  | Sensitivity, % | Specificity, % | P values |
| All cases | 81.08 (30/37) | 95.83 (46/48) |  |
| Cases with different stages |  |  | All P >0.05 |
| I | 80.00 (8/10) | 95.83 (46/48) |  |
| II | 83.33 (10/12) | 95.83 (46/48) |  |
| III-IV | 80.00 (12/15) | 95.83 (46/48) |  |
| Cases with different histological types |  |  | All P >0.05 |
| AC | 80.95 (17/21) | 95.83 (46/48) |  |
| SCC | 81.25 (13/16) | 95.83 (46/48) |  |
| ^*^ cancer-free subjects include 22 healthy subjects and 26 patients with chronic obstructive pulmonary disease. | | | |
| Abbreviations: NSCLC, non-small-cell lung cancer; AC, adenocarcinoma; SCC, squamous cell carcinoma. | | | |

| **Additional Table 4.** Association of the snoRNA expressions with demographic characteristics of the NSCLC patients | | | |
| --- | --- | --- | --- |
|  | SnoRNA, OR (95% CI), P* | | |
| Characteristics | SNORD33 | SNORD66 | SNORD76 |
| Age | 0.39 (0.22 to 1.25), 0.46 | 0.63 (0.34 to 0.95), 0.53 | 0.53 (0.48 to 1.87), 0.48 |
| Gender | 1.32 (0.67 to 3.02), 0.38 | 1.25 (0.58 to 3.12), 0.37 | 1.31 (0.57 to 2.68), 0.49 |
| Ethnic group | 1.28 (0.85 to 2.95), 0.23 | 0.68 (0.36 to 1.27), 0 54 | 1.45 (0.62 to 2.77), 0.46 |
| Smoking status | 1.61 (0.56 to 3.08), 0.57 | 1.84 (0.61 to 3.33), 0.69 | 1.46 (0.58 to 3.13), 0.67 |
| Abbreviations: NSCLC, non-small-cell lung cancer. OR = odds ratio; CI = confidence interval. | | | |
| * Uunivariate analysis. All P values are two-sided, and P≤0.05 was considered statistically significant. | | | |
